# 

**Published:** 1892-10

**Authors:** 


### CONTENTS:

ORIGINAL ARTICLES: PAGE

Strongylus Armatus. By J. F. Winchester, D. V. S. .... 579
United States Veterinary Medical Association .... 609
Southern Cattle Plague. By Frank S. Billings .... 613
Earth Worms and the Bacilli of Tuberculosis .... 629
Tuberculosis of Cattle. By Leonard Pearson, B. S., V. M. D. .... 630

SOCIETY PROCEEDINGS:

Veterinary Medical Association of New Jersey .... 639
Pennsylvania State Veterinary Medical Association .... 641

BOOKS AND PAMPHLETS RECEIVED .... 642

